# Diagnosing Kearns-Sayre syndrome requires documentation of the underlying genetic defect

**DOI:** 10.11604/pamj.2025.50.92.37174

**Published:** 2025-04-02

**Authors:** Josef Finsterer, Sinda Zarrouk

**Affiliations:** 1 Neurology and Neurophysiology Center, Vienna, Austria,; 2 Genomics Platform, *Institut Pasteur de Tunis (IPT)*, Tunis-Belvédère, Tunis, Tunisia

**Keywords:** Mitochondrial disorder, Kearns-Sayre syndrome, respiratory chain, retinopathy, vision

## To the Editors of the Pan African Medical Journal

We read with interest the article by Ennejjar *et al*. about a 9-year-old male with Kearns-Sayre syndrome (KSS) diagnosed during ophthalmologic school-based screening and muscle biopsy findings [1]. The diagnosis was established upon documentation of a “salt and pepper retinopathy” without genetic confirmation [1]. Because the patient presented with an atrio-ventricular block-III, a pacemaker was implanted [1]. The study is appealing but carries limitations that raise concerns and should be discussed.

The main limitation of the study is that the diagnosis of KSS was not genetically confirmed. KSS is, in the vast majority of cases, due to single mtDNA deletions and only rarely due to mtDNA point mutations (e.g. m.3243A>G) [2]. Documentation of the genetic background is crucial because genetic counselling strongly depends on the molecular basis of the syndrome. In four percent of the cases, KSS is inherited via the maternal line, but in the reminder single mtDNA deletions causing KSS occur sporadically, probably at the germ-cell level, and are not inherited [3]. We should be told if the index patient´s mother had undergone neurological exam and if she carried an mtDNA deletion or phenotypic features of KSS.

We disagree with the statement that KSS is diagnosed upon muscle biopsy [1]. Muscle biopsy findings in KSS may be non-specific and similar to those in other mitochondrial disorders, which is muscle biopsy is not diagnostic for KSS. Muscle biopsy may be only useful for collecting myocytes and for mtDNA extraction. mtDNA deletions are usually more effectively found in muscle tissue than in blood lymphocytes.

We disagree with the statement in the abstract that ptosis and ophthalmoparesis are ophthalmologic manifestations of KSS [1]. Ptosis and ophthalmoparesis are due to muscle weakness and, therefore clearly neurological manifestations of the disease. Mitochondrial myopathy is usually the reason why creatine kinase (CK) is permanently elevated in KSS patients. Frequently, limb muscles are affected in KSS.

Patients with KSS may not only experience atrio-ventricular blocks but also ventricular arrhythmias [4]. Ventricular arrhythmias may not only be due to conduction disturbances, but also due to heart failure resulting from dilative cardiomyopathy [5]. Therefore, it is crucial to know the results of echocardiography, if pro-brain natriuretic peptide (pro-B-type natriuretic peptide (proBNP)) was elevated, and if long-term electrocardiogram (ECG) recordings revealed ventricular arrhythmias (e.g. ventricular tachycardia). Documentation of ventricular arrhythmias may be an indication for implantation of an implantable cardioverter defibrillator (ICD) rather than a pacemaker, as in the index patient.

Another limitation of the study is that no follow-up data were presented. It would be interesting to know for how long the patient survived and if the course remained stable or was progressive. Did the patient experience clinical manifestations during the course other than the ones described?

Overall, the interesting study has limitations that call the results and their interpretation into question. Clarifying these weaknesses would strengthen the conclusions and could improve the study. Diagnosing KSS requires genetic confirmation.
